# Ethical Considerations and Fundamental Principles of Large Language Models in Medical Education: Viewpoint

**DOI:** 10.2196/60083

**Published:** 2024-08-01

**Authors:** Li Zhui, Li Fenghe, Wang Xuehu, Fu Qining, Ren Wei

**Affiliations:** 1 Department of Vascular Surgery The First Affiliated Hospital of Chongqing Medical University Chongqing China

**Keywords:** medical education, artificial intelligence, large language models, medical ethics, AI, LLMs, ethics, academic integrity, privacy and data risks, data security, data protection, intellectual property rights, educational research

## Abstract

This viewpoint article first explores the ethical challenges associated with the future application of large language models (LLMs) in the context of medical education. These challenges include not only ethical concerns related to the development of LLMs, such as artificial intelligence (AI) hallucinations, information bias, privacy and data risks, and deficiencies in terms of transparency and interpretability but also issues concerning the application of LLMs, including deficiencies in emotional intelligence, educational inequities, problems with academic integrity, and questions of responsibility and copyright ownership. This paper then analyzes existing AI-related legal and ethical frameworks and highlights their limitations with regard to the application of LLMs in the context of medical education. To ensure that LLMs are integrated in a responsible and safe manner, the authors recommend the development of a unified ethical framework that is specifically tailored for LLMs in this field. This framework should be based on 8 fundamental principles: quality control and supervision mechanisms; privacy and data protection; transparency and interpretability; fairness and equal treatment; academic integrity and moral norms; accountability and traceability; protection and respect for intellectual property; and the promotion of educational research and innovation. The authors further discuss specific measures that can be taken to implement these principles, thereby laying a solid foundation for the development of a comprehensive and actionable ethical framework. Such a unified ethical framework based on these 8 fundamental principles can provide clear guidance and support for the application of LLMs in the context of medical education. This approach can help establish a balance between technological advancement and ethical safeguards, thereby ensuring that medical education can progress without compromising the principles of fairness, justice, or patient safety and establishing a more equitable, safer, and more efficient environment for medical education.

## Introduction

Large language models (LLMs), such as OpenAI's ChatGPT series, Microsoft's Copilot, and Google's Gemini, are sophisticated artificial intelligence (AI) tools that are trained on vast text datasets via deep learning techniques. These models exhibit the ability to generate language that is highly similar to human speech; thus, their use in medicine is becoming increasingly widespread. From assisting scientific research to informing clinical decisions and supporting medical education, LLMs can be used to optimize the use of information and resources across diverse domains [1].

In the technology-driven transformation of medical education, the introduction of LLMs has the potential to revolutionize traditional learning and teaching methods. These advanced language models can significantly enhance the personalization and practicality of education by generating tailored learning materials and simulating digital experiences that resemble real clinical scenarios [2]. LLMs dynamically generate customized learning materials and assessments based on students' progress and needs, thereby improving the efficiency and effectiveness of learning [3]. Moreover, by simulating patient interactions, LLMs can help students practice and hone their diagnostic and communication skills in a safe environment, which is a crucial element of attempts to nurture the practical abilities of medical professionals [4].

Biomedical ethics is founded on 4 fundamental principles: autonomy, nonmaleficence, beneficence, and justice [5]. Medical education should not only aim to cultivate these principles among students but also continuously evolve and improve under their guidance [6]. Due to the increasingly widespread use of LLMs in medical education research, some foreseeable ethical issues present challenges to these basic principles. For example, the privacy and data security risks associated with LLMs can infringe upon individuals' autonomy to control their own information, thus potentially causing harm. Biases in training data and algorithms can lead to unfair diagnostic and treatment decisions that can harm patients, and these biases may also risk spreading misinformation, thereby violating the principle of beneficence. Additionally, issues related to academic integrity and concerns regarding responsibility and copyright may risk violating the principle of justice, among others.

While previous research on education-related LLMs has emphasized the tasks of enhancing key competencies or exploring educational LLM programs and related concepts, it has failed to account for ethical principles specifically in the development and application of such technologies [7,8]. Addressing and resolving these issues are vital not only for technological advancement but also for the tasks of ensuring high-quality medical education and protecting the rights of both practitioners and patients. Therefore, this viewpoint article aims to explore and analyze the potential ethical issues that LLMs might raise in the context of future medical education and to identify fundamental principles for developing an ethical framework for these technologies as well as corresponding methods for their implementation.

### Ethical Concerns Related to the Development of LLMs

#### Privacy and Data Risks

In the era of electronic information technology, privacy risks and data security have always been associated with advancements in information technology. LLMs are associated with enormous numbers of parameters and training data sets and may “memorize” sensitive personal information from their training data, thus, enabling these models to generate information that contains specific individual data [9]. Jegorova et al [10] confirmed the risk of privacy leakage during the inference process exhibited by learning models. When teachers or educational institutions use data, including students' personal information, social background information, and health data, to train models to provide personalized feedback regarding students’ learning interests and progress, this process entails the risk of sensitive information leakage. When LLMs are used for the purpose of assisted learning, such as in the contexts of clinical case discussions, tasks involving sensitive patient data such as name, sex, age, medical history, or final diagnoses, and even imaging data such as computed tomography scans and magnetic resonance imaging for assistance in interpretation, patient privacy is put at risk. Previous studies have shown that even if this information is anonymized when it is input, some advanced models can reidentify such personal information from large data sets through so-called linkage attacks, thus, resulting in information exposure [10]. Rocher et al [11] reported that LLMs could use as few as 15 demographic attributes to reidentify 99.98% of personal data in any anonymized data set. Therefore, deidentifying information alone is insufficient to protect patient privacy, a situation which poses tremendous challenges with regard to protecting patient privacy when LLMs are used in the context of medical education. Furthermore, as the training and user data of LLMs are stored on cloud servers, any security vulnerabilities could result in the leakage of such sensitive information [12]. LLM development companies, such as OpenAI, may use users' personal information for service analysis, improvement, or research purposes and retain the right to share users' personal information with third parties without explicit user consent [13]. All of these factors exacerbate the risks pertaining to privacy and data security in medical education processes.

#### AI Hallucination

Clinical practice guidelines and consensus documents, which are continually updated, are significantly valuable as references in the context of medical education. However, “AI hallucinations” on the part of LLMs represent a major obstacle to the effective use of these documents as educational content. In the field of medicine, AI hallucinations occur when LLMs generate responses that appear to be logical but are actually incorrect, inconsistent, or fabricated, including those that rely on fabricated data and forged references [14]. Amir et al [15] compared ChatGPT 3.5 and Google BARD in terms of their ability to answer questions related to lung cancer, revealing that these LLMs exhibited error rates ranging between 17.5% and 27.5%. Similar issues have been observed in the field of dentistry, in which context LLMs can offer vague, outdated, and inaccurate information [16]. Studies have highlighted accuracy concerns with regard to LLMs’ responses in medical contexts [17,18]. Several factors contribute to AI hallucinations in the field of medicine. First, LLMs' training data, which are sourced mostly from the internet, include outdated and inaccurate information that lacks quality control. Second, access to authoritative databases such as PubMed, UpToDate, and Cochrane requires subscriptions, thus, restricting LLMs from obtaining up-to-date and reliable research data [19,20]. Additionally, limitations regarding LLMs’ reasoning and probabilistic text generation capabilities exacerbate the problem of AI hallucination.

Given the high level of precision required in medicine, even minor errors can have catastrophic consequences for patients. When medical educators use unverified LLM-generated content, the “one-to-many” model facilitates the widespread propagation of erroneous information. Medical students, who are in the process of acquiring foundational knowledge and trust their educators, are especially vulnerable to such misinformation. This issue can severely impact their future clinical decisions. The use of inaccurate LLM content to evaluate students reinforces incorrect information, leading to flawed assessments and continued learning on the basis of faulty knowledge. In academic research, students may unknowingly cite false references or use incorrect data, resulting in academic misconduct. If erroneous LLM-generated information is widely disseminated in medical education, it can lead to systemic errors in clinical decision-making, thus, endangering patient health and safety. This situation undermines the rigor of medical education, erodes public trust in health care, and raises ethical issues and challenges related to academic integrity, which ultimately impact the professionalism and social responsibility of the medical field.

#### Training Data and Algorithmic Biases

Zack et al [21] reported that ChatGPT-4 often resorts to stereotypes and exhibits bias toward specific races, ethnicities, and sexes in terms of its diagnostic outputs. Additionally, treatment suggestions have been shown to be linked to demographic factors, to favor costlier procedures and to exhibit variations in terms of patient perceptions [21]. This widespread problem of unfair responses is primarily due to such LLMs’ training data and algorithmic biases.

Bias in training data is the most direct cause of bias in LLMs’ responses. LLMs learn from vast amounts of unfiltered text; thus, if these data contain biases related to sex, race, culture, or socioeconomic background, the model replicates these biases. A study on a chest x-ray classifier that had been trained mainly on White patients revealed that it lacked recognition capability in cases involving non-White patients [22]. As the amount of software-generated data increases, such information may be used as training data, further reinforcing the original biases [23].

Algorithmic bias on the part of LLMs exacerbates the problem of output bias. During the process of LLM design, the optimization goals and evaluation criteria used often focus on the majority population, neglecting the needs of the minority. For example, if an LLM is trained primarily on Western cases of alcoholic cirrhosis without considering geographic and cultural diversity or including data concerning hepatitis B-induced cirrhosis from other regions, it may provide inaccurate guidance regarding the diagnosis and treatment of hepatitis B-related cirrhosis. Failure to address training data bias and adjust algorithms perpetuates unfairness in terms of clinical practices [24]. Additionally, developers may intentionally introduce biases, which can affect data processing, model training, algorithm selection, and application, ultimately causing specific groups to receive unfair treatment. Algorithm bias impacts LLMs’ responses regardless of the presence of training data bias [25].

Biases embedded in data and algorithms are reproduced in LLMs’ outputs, resulting in incomplete educational content and depriving students of comprehensive and in-depth learning guidance. This lack of diversity in educational content deprives students of a balanced medical perspective, which impacts the quality and fairness of education. Future doctors may lack the knowledge and ethical sensitivity they need to serve diverse patient populations effectively, potentially leading to misdiagnoses or inappropriate treatments. Biased information based on stereotypes and unfair descriptions of specific groups can distort students' ethical concepts during the process of diagnosis and treatment, inadvertently fostering biases against these groups. Furthermore, this gap not only affects individual students' achievements but is also amplified through medical education, thereby weakening the health care system's ability to meet the needs of diverse patient populations. LLM biases violate core biomedical ethics principles, such as nonmaleficence, beneficence, and justice. Ensuring fair and neutral LLM use in medical education is crucial with regard to efforts to maintain educational quality and uphold professional ethics.

#### Deficiencies in Transparency and Interpretability

When users ask questions, LLMs usually generate a predefined response. However, users often cannot comprehend how this response is generated or the underlying logic. This issue is known as the “black box” effect, in which context transparency and interpretability are lacking [26]. Transparency refers to whether the model's operation and decision-making process can be presented clearly to the user, while interpretability refers to whether users can understand the logic and reasons that underlie the decisions or text, thus, generated [9]. Such a lack of transparency and interpretability is mainly due to LLMs’ large model structures and complex training data, which make their internal operations difficult to understand.

In the context of medical education, the lack of transparency and interpretability in LLMs can prevent teachers and students from assessing the accuracy of information effectively [27]. If teachers convey inaccurate information to students, such information inevitably entails risks to patient safety in clinical practice. The fact that students exhibit a high level of trust in their teachers indicates that these deficiencies can have more widespread and serious impacts than in other fields. Additionally, a lack of understanding of the logic underlying LLMs may lead to rigid thinking on the part of students, who may neglect the importance of logical thinking in medical education, thus weakening efforts to cultivate their critical thinking. Logical reasoning is crucial in clinical practice with regard to analyzing conditions accurately and formulating treatment plans. A lack of logical reasoning on the part of doctors may lead to misdiagnosis, mistreatment, and risks to patient safety. Moreover, when teachers cannot grasp the logic employed by LLMs, they may convey conclusions to students without explaining the corresponding deductive process, thus hampering their ability to guide students and decreasing the quality of teaching.

### Ethical Concerns Related to the Application of LLMs

#### Emotional Intelligence Deficiency

Although LLMs are trained to provide empathetic responses in the context of engaging with patients [28]—recent research has shown that ChatGPT even exhibits higher levels of empathy when consulting on systemic sclerosis than do neurologists [29]—the artificial empathy expressed by AI generally cannot replicate the subtle genuineness of the emotions conveyed by health care professionals [30]. True emotional expression is the result of perceiving and understanding the patient's emotions through real-time communication, especially by sensing changes in tone, facial expressions, and body language, with the goals of comprehending the patient’s needs and concerns and providing personalized feedback. However, existing LLMs cannot perceive these nonverbal emotional changes. Furthermore, the expression of empathy by LLMs is limited to textual descriptions of emotions, whereas variations in tone, facial expressions, and body language on the part of health care professionals, such as handshakes or hugs, often convey empathy more effectively than can words alone [1]. Prof Marc Succi suggested that excessive reliance on artificial emotional communication in medical settings may exacerbate the societal public health issue of “loneliness” [31]. Establishing and developing empathy involves addressing the complexity and uncertainty that characterize real interactions, which are shaped by patients' diverse backgrounds, beliefs, and values. LLMs, as teaching tools, cannot convey a diverse range of emotional expressions beyond the level permitted by text, thus, preventing medical students from gathering materials that can help them improve their communication skills and empathy through such interactions. In addition to direct patient communication, teachers who exhibit empathy and care in clinical settings also serve as good examples who can enhance students' empathy. Sole reliance on LLMs for tasks such as medical history collection, condition disclosure, and obtaining consent can reduce the presence of empathy in clinical environments, thus, conflicting with the goals of medical ethics education. Excessive reliance on artificial emotional intelligence can lead to a lack of empathy among medical students. The development of empathy requires ongoing practice in diverse emotional settings, which static LLMs cannot provide.

#### Educational Unfairness

Unfairness in the context of traditional medical education is characterized primarily by several aspects: uneven resource distribution, disparities in faculty strength, high tuition fees that limit student access, and geographical disparities in terms of school location [32]. While LLMs offer rich educational resources and opportunities, their application is a double-edged sword with respect to educational fairness.

For institutions that have only limited teaching resources, LLMs can aggregate rich teaching resources at a global level, thus, providing students with high-quality educational experiences. They also offer relatively equal learning opportunities to regions located in remote geographical areas and enable nonnative speakers to access and understand medical knowledge in other languages through cross-language support [33]. However, medical schools that have better technological infrastructure, higher budgets, and access to the most up-to-date technologies may find it easier to take advantage of the benefits of LLMs in the context of education. In contrast, schools and regions with limited resources may experience exacerbated resource allocation inequalities because of their inability to afford these technologies, their lack of the professional knowledge necessary to implement these technologies, and restrictions on accessing LLMs (such as ChatGPT, which is currently unavailable to Chinese users). Moreover, the use of LLMs requires extensive data processing, which may expose data to privacy and security risks. Institutions that lack sufficient resources and robust infrastructure may struggle to safeguard user data, thus, increasing the risk of data leakage and misuse. With respect to academic achievements, individuals who can use these tools may gain access to more recognition and opportunities within the academic community, whereas students and researchers who lack such access may face disadvantages in terms of academic competition [34].

#### Academic Integrity Concerns

Issues related to academic integrity in the context of medical education notably include plagiarism and cheating. LLMs greatly facilitate medical research by summarizing published articles and assisting with data analysis or literature retrieval. Research has indicated that when abstracts generated by LLMs and those authored by humans are presented simultaneously, university professors struggle to distinguish between them [35]. Another study that examined LLMs' generation of fictitious article abstracts revealed that peer reviewers could identify only 68% of abstracts generated by ChatGPT as fictitious [36]. This outcome indicates that the quality of the text produced by LLMs has approached or reached a professional level, thus, giving rise to opportunities for academic plagiarism; namely, students may use LLMs to generate abstracts, data, or even complete draft papers. Despite the originality of the content generated by LLMs, students who submit these texts as their own work may be suspected of plagiarism. With regard to one recently published article, the direct replication of generated content, including the prompts from LLMs—the regenerated response—resulted in article retraction [37]. Numerous other articles now face similar scrutiny, and the question of whether these will be retracted by the journal or revised remains undecided [38,39]. This excessive reliance and lack of critical use of LLMs have caused serious damage to academic integrity and posed ethical challenges. Furthermore, the integration of LLMs into browsers facilitates direct searches for source texts or other multimedia documents, which can exacerbate issues related to plagiarism and make them more prevalent when teachers or students use these data directly. A well-known test of the accuracy of LLMs in the context of medical questions is ChatGPT's successful passing of the United States Medical Licensing Exam [40]. Many studies have shown that LLMs achieve high scores in various disciplines [41-43]. This high level of accuracy enables students to use LLMs to cheat on their examinations. Excessive reliance on LLMs may decrease students' research ability, critical thinking, innovation, and motivation to engage in proper learning [44]. This situation can perpetuate students’ reliance on LLMs and academic misconduct, thus, leading to a vicious cycle. Additionally, if students and teachers plagiarize LLM-generated content without critical evaluation, erroneous or biased information may become widespread.

#### Responsibility and Copyright Concerns

In the context of traditional clinical education, clinical teachers supervise students' operations, provide students with guidance, and take responsibility for adverse clinical events, while students adhere to medical standards and ethical norms. Medical institutions must ensure a safe learning environment and sufficient supervision. This clear division of responsibilities can help protect patient safety and improve the quality of education.

In the future, the introduction of LLMs into medical education will inevitably add new dimensions to the division of responsibilities. From a legal perspective, AI lacks the legal standing of a human being, thus leaving humans as the ultimate accountable parties [45]. However, in medical settings, the responsibility for patient harm due to LLM biases and inaccuracies is unclear, a situation which affects doctors, patients, and institutions such as medical facilities and developers. LLM developers should ensure that their tools are accurate and safe, but entities such as OpenAI disclaim responsibility for the texts generated by their LLMs [46]. Existing ethical and legal frameworks have not fully adapted to the challenges posed by such emerging tools, thus, leading to ambiguity in terms of responsibility attribution and regulation in the context of actual application.

When teachers use LLMs to generate teaching outlines, lecture notes, or textbooks or when students use LLMs to write academic papers—especially when LLMs access original texts and images from the internet through search engines—and then copy from copyrighted source materials directly or borrow from them excessively, copyright disputes can occur [4]. To protect copyrights and maintain academic integrity more effectively, current guidelines issued by the International Committee of Medical Journal Editors and the Committee on Public Ethics suggest that LLMs such as ChatGPT should not be listed as authors of papers [47]. LLMs cannot bear the same responsibility as human authors or provide substantive explanations for the content they generate. However, this approach is not universally accepted, and the role of LLMs in authorship declarations remains the subject of ongoing discussion by various publishing institutions [48,49].

### AI-Related Laws and Ethics

#### Overview

Due to the advancement of AI technology in various domains, particularly with regard to the development of LLMs, multiple countries and international organizations have actively formulated or updated ethical and legal guidelines. These measures focus on key areas such as privacy protection, transparency, algorithmic fairness, and accountability and offered reliable guidance concerning the reasonable use and development of artificial intelligence technologies. AI legal and ethical principles are in Multimedia Appendix 1.

#### Laws

The General Data Protection Regulation of the European Union, which was enacted in 2018, offers stringent guidelines for handling sensitive data in the education and health care sectors. These guidelines emphasize the pseudonymization or anonymization of personal data storage, requiring the highest privacy settings, and ensuring transparency in terms of data processing. Additionally, they strengthen users’ control over their personal information, thus, empowering individuals to manage their data effectively [50]. In China, the Personal Information Protection Law, which was implemented in 2021, establishes rigorous standards for the protection of personal information, particularly with regard to user awareness and consent rights [51]. Similarly, the Health Insurance Portability and Accountability Act in the United States prioritizes the protection of medical information and respect for patient privacy [52].

#### Ethical Guidelines and Initiatives

International guiding principles have extensively discussed issues pertaining to transparency, interpretability, algorithmic fairness, and accountability in the context of AI. The Institute of Electrical and Electronics Engineers Global Initiative on Ethics of Autonomous and Intelligent Systems, which was founded in 2019, emphasized the transparency of autonomous systems in AI design processes and ethical guidelines for handling private data and algorithmic biases [53]. The European Union's White Paper on Artificial Intelligence, which was released in February 2020, elaborated on ethical frameworks for system transparency, privacy data, and algorithmic biases, particularly with regard to the importance of avoiding bias and discrimination and ensuring the fair use of AI decision support systems across diverse populations [54]. The United Nations Educational, Scientific and Cultural Organization's “Recommendations on the Ethics of Artificial Intelligence,” which was issued in November 2021, systematically addressed privacy rights, data protection, transparency, interpretability, responsibility, and accountability in the context of AI applications, emphasizing fairness and nondiscrimination [55]. In January 2024, the World Health Organization issued the “Ethics and Governance of AI in Health: Guidance on Large Multimodal Models,” which was the first ethical standard for large multimodal models such as LLMs. This document emphasized transparency, interpretability, and accountability; promoted fair access; and prioritized inclusivity [56]. In March 2024, the United Nations General Assembly adopted a US-led resolution titled “Seizing the opportunities of safe, secure and trustworthy artificial intelligence systems for sustainable development,” which represented the first global consensus on AI governance. This resolution addressed the needs to bridge regional AI gaps, protect against discrimination and bias, safeguard privacy, and respect intellectual property (IP) rights, among other 13 proposals [57].

#### Limitations

Existing legal and ethical guidelines ensure the safe, transparent, and fair use of AI systems. However, when LLMs are used in the context of medical education, significant limitations emerge. First, these guidelines, which have been shaped by various national, organizational, and cultural contexts, reflect different social values, historical backgrounds, and priorities. For instance, the General Data Protection Regulation and the Personal Information Protection Law in China emphasize data privacy, whereas the Institute of Electrical and Electronics Engineers guidelines prioritize technological innovation. Such differences can lead to conflicts and increase the complexity and uncertainty of applying LLMs across different countries. Second, despite the fact that these guidelines emphasize transparency and interpretability, the complex algorithms and nonlinear structures of LLMs make these principles difficult to achieve. Existing guidelines lack detailed, actionable recommendations that can help users understand and trust LLM decision-making in the context of medical education. Furthermore, LLMs may amplify biases in their training data, thus, leading to unfair outcomes. While many guidelines have emphasized the importance of avoiding discrimination and bias, they have not provided specific tools or methods to detect and correct biases within LLMs. Additionally, current ethical oversight and accountability mechanisms are inadequate with respect to the use of LLMs in medical education. When LLMs generate misleading or inaccurate content that could cause harm, existing frameworks lack clear guidelines for determining when human intervention is necessary, how to monitor and adjust the model's output, and how to establish accountability. Most importantly, existing legal and ethical guidelines in the field of medical education are fragmented and lack cohesiveness. They do not comprehensively address unique challenges such as academic misconduct, plagiarism, and copyright issues. Consequently, educators often struggle to find clear, authoritative solutions in practice. Therefore, it is critical to develop an ethical framework that is specifically tailored to the application of LLMs in medical education, thereby ensuring the safety, efficacy, and fairness of such technologies and promoting their widespread and in-depth application in this critical field.

#### Fundamental Principles of and Ways of Implementing LLM Ethics in Medical Education

#### Overview

**Figure 1:**
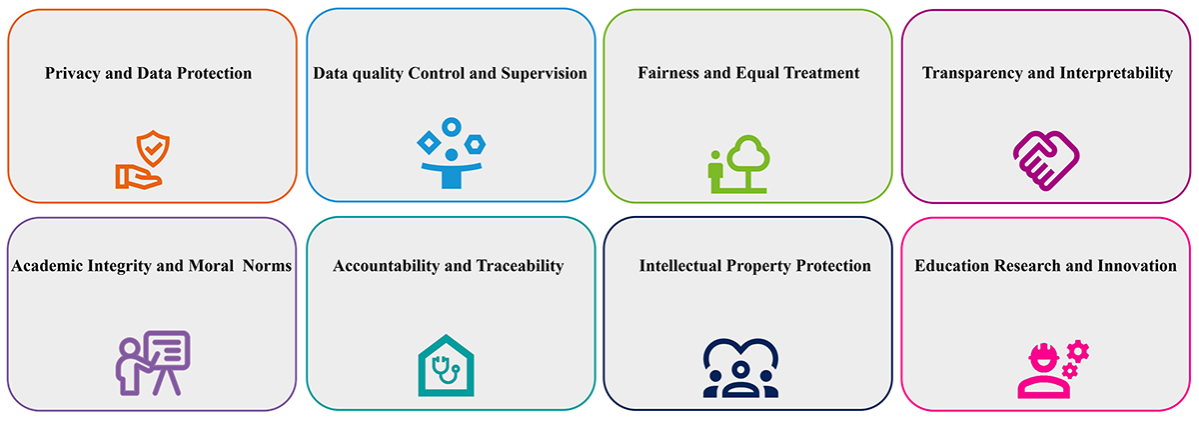
Eight fundamental principles regarding the use of large language models in medical education.

To ensure that LLMs are used within appropriate legal and ethical frameworks, it is essential to establish a unified ethical framework. In light of the broad biomedical principles proposed by Beauchamp and Childress, namely, beneficence, justice, autonomy, and nonmaleficence [5], we propose fundamental principles and implementation methods for the use of LLMs in the context of medical education that are in line with these biomedical ethical principles (Figure 1 and Table 1).

**Table 1:** Implementation methods aligned with biomedical ethical principles regarding the use of large language models (LLMs) in medical education.

Ethical principles for LLMs in medical education	Implementation methods	Biomedical ethical principles
Privacy and data protection	Multilayered privacy protection mechanisms Data encryption and secure storage Strict data usage policies Strict access controls	Nonmaleficence and autonomy
Data quality control and supervision	Develop independent LLM databases Link to authoritative databases Maintain a critical approach	Beneficence and justice
Fairness and equal treatment	Diverse and comprehensive training data Fairness review and monitoring mechanisms Open and accessible resources and tools Cross-regional and cross-economic background collaboration	Justice
Transparency and interpretability	Model documentation and operational guidelines Implement interpretative interfaces or tools Regular public evaluations and feedback	Autonomy
Academic integrity and moral norms	Establish policies and guidelines Develop and use detection tools Maintain educators' leadership roles Foster empathy and compassion	Nonmaleficence and beneficence
Accountability and traceability	Clear responsibility framework Detailed logging and monitoring systems Independent review and investigation committees Clear complaint and feedback channels	Nonmaleficence and justice
Intellectual property protection	Strict IP compliance processes Copyright statements and terms of use Monitoring and detection systems Complaint and feedback mechanisms	Justice and autonomy
Educational research and innovation	Establish dedicated research and development funds Creating an open teaching experimentation environment Optimizing teaching content	Beneficence

#### Privacy and Data Protection

To mitigate the risk of sensitive data breaches effectively and ensure both data security and privacy protection, it is crucial to implement a series of robust data protection measures involving dedicated LLM training databases that are specifically designed for medical education.

Multilayered Privacy Protection Mechanisms: Advanced data deidentification techniques, such as generalizing and suppressing sensitive information, must be used to prevent reidentification. Differential privacy techniques must be employed to enhance protection by adding noise to the data [58].

Data Encryption and Secure Storage: Data encryption and storage must be enhanced based on tamper-proof hardware security modules and distributed storage systems with the goals of preventing attacks and ensuring data security in terms of both storage and transmission [59].

Strict Data Usage Policies: Strict policies must be established and followed to ensure that data providers have control over and transparency regarding their data use. Users must be informed about data usage and allowed to withdraw their consent at any time.

Strict Access Controls: Multifactor authentication and stringent access controls, such as SMS text messaging verification and facial recognition, must be implemented to ensure that only authorized personnel access sensitive data [60].

#### Data Quality Control and Supervision

To prevent partial and inaccurate responses based on erroneous or outdated training data, effective quality control and supervision mechanisms must be established.

Develop independent LLM databases: all training data should be reviewed by clinical experts before incorporation to ensure authenticity and accuracy. LLM-generated content must be regularly reviewed and evaluated to identify and correct biases and errors promptly.

Link to authoritative databases: authoritative databases such as PubMed and UpToDate must be used to ensure the accuracy and relevance of training data. These resources are to be updated regularly with the latest medical research, thereby reducing errors and biases as well as enhancing data credibility.

Maintain a critical approach: educators and students should assess LLM-generated information critically by verifying it by reference to authoritative databases and other language models. This practice can ensure high-quality standards in medical education involving LLMs.

#### Fairness and Equal Treatment

Mitigating biases in LLMs can ensure that all students and educators have equal opportunities and resources in the context of medical education, thereby eliminating the health disparities and knowledge inequalities caused by these biases.

Diverse and comprehensive training data: developers must use diverse datasets during model training to avoid biases. This process involves reviewing and filtering data with the goal of eliminating the factors that lead to discrimination or unfair treatment.

Fairness review and monitoring mechanisms: fairness review and monitoring mechanisms must be incorporated into the model's deployment and usage. The biases or unjust tendencies exhibited by the model outputs must be evaluated and detected regularly, and adjustments and optimizations must be made as needed.

Open and accessible resources and tools: open and accessible resources and tools must be provided to students and educators at various economic levels and geographic locations to enable them to use LLMs on an equal basis. For example, developing lightweight or localized versions of a model can support advanced technology adoption in resource-limited areas.

Cross-regional and cross-economic background collaboration: cross-regional and cross-economic collaboration platforms and communities must be established and supported with the goal of promoting the sharing of knowledge and resources, thereby mitigating the educational inequalities caused by regional disparities.

#### Transparency and Interpretability

Ensuring that the operations and decision-making processes of LLMs are transparent and explainable to students and educators is critical with respect to enhancing students' and educators’ understanding and trust in the system.

Model documentation and operational guidelines: developers should provide comprehensive documentation and guidelines to explain the model's mechanisms, input-output processes, and decision-making logic. These guidelines should cover design objectives, the sources of training data and corresponding methods, and potential limitations with the aim of helping users understand the model's construction and functionality.

Interpretative interfaces or tools: in the context of model use, interpretative interfaces or tools must be implemented to allow students and educators to understand the model's reasoning and decisions in real time. For example, visual tools can reveal how the model processes input data and generate outputs, thereby enhancing the model’s transparency and interpretability [61].

Regular public evaluations and feedback: involving experts and users in regular evaluations and reviews and establishing effective feedback mechanisms can help identify and correct issues or biases associated with the use of the model, thereby promoting continuous improvement and optimization.

#### Academic Integrity and Moral Norms

An emphasis on academic integrity and ethical norms can prevent students and educators from gaining undue advantages from LLMs or from engaging in misconduct, such as plagiarism or cheating. Students and educators should use LLMs reasonably, avoid excessive reliance on such technologies, and focus on cultivating empathy, compassion, and critical thinking.

Establish policies and guidelines: educational institutions should establish specific LLM usage policies and guidelines based on a unified set of ethical principles to specify the legal and ethical use of LLMs as well as to prohibit any form of academic misconduct clearly and explain the corresponding consequences.

Develop and use detection tools: detection tools must be developed to identify and prevent inappropriate content or plagiarism with regard to LLM-generated material, thus ensuring that students' submissions and research results are genuine and original [62].

Maintain educators' leadership roles: during the teaching process, educators should continue to play a leadership role by encouraging students to analyze and evaluate LLM-generated content critically and emphasizing that models are auxiliary tools rather than substitutes for human thought. Educators should encourage students to question and verify information from LLMs, thereby enhancing their independent thinking skills.

Foster empathy and compassion: in clinical practice, cultivating students' empathy and compassion through rich interpersonal interactions can enhance the humanistic aspect of patient care. Educators should lead by example, such as by demonstrating material and guiding students in the process of applying these skills in real clinical settings. This approach can help students understand and respect patients' emotions and needs, ultimately improving the overall quality of health care services.

#### Accountability and Traceability

To ensure that responsibilities and accountability procedures are defined clearly when LLM decision errors negatively affect patient health, clear accountability and traceability mechanisms must be established.

Clear framework for responsibility: developing a clear framework of responsibilities and obligations for model developers, deployers, and users at different stages is crucial. Developers should ensure that the model's design and training meet safety and fairness standards; deployers must ensure compliance with supervision and quality control measures; and users must make and verify professional judgments on the basis of the model's recommendations.

Detailed logging and monitoring systems: implementing detailed logging and monitoring during the use of LLMs involves recording the model's inputs, outputs, and decision paths, which can make it possible to trace the decision-making process when issues arise.

Independent review and investigation committees: independent committees must be established to address and investigate health issues resulting from LLM decision errors while ensuring fair procedures. These committees should include technical experts, legal advisors, and medical professionals who can assess responsibilities jointly and recommend improvements.

Clear complaint and feedback channels: providing clear complaint and feedback channels for patients and users allows them to report issues and seek help promptly in cases involving harm.

#### Intellectual Property Protection

Ensuring compliance with IP laws during the use of LLMs and protecting the rights of knowledge creators and holders are critical to the task of preventing unauthorized knowledge use and infringement.

Strict IP compliance processes: strict IP compliance processes must be established to ensure that LLMs use only authorized or publicly licensed data. Developers should document data sources meticulously and review them regularly for legality and compliance.

Copyright statements and terms of use: when LLM-generated content is used, clear copyright statements and terms of use must be provided. Such statements and terms of use can ensure that users understand and comply with IP laws, thus preventing the unauthorized creation or dissemination of protected content.

Monitoring and detection systems: effective monitoring and detection systems can be implemented to identify and prevent unauthorized use and potential infringement. For example, copyright detection tools can automatically flag potentially infringing content in model outputs [63].

Complaint and feedback mechanisms: complaint and feedback mechanisms can be established to allow creators and IP holders to report and address potential infringement issues promptly, thus facilitating efficient remedial actions.

#### Educational Research and Innovation

Taking advantage of the benefits of LLM technology for educational research and innovation can enhance the quality and effectiveness of medical education, thus promoting the continuous improvement and optimization of teaching methods and content.

Establish dedicated research and development funds: dedicated research and development funds must be established to support the attempts of educational institutions and academic groups to conduct LLM-centered innovative educational projects and research. Encouraging interdisciplinary collaboration and integrating the most recent advancements in AI and medical education can facilitate the exploration of novel teaching methods and content.

Open teaching experimentation environment: establishing an open experimentation and testing environment can allow educators to explore innovative teaching methods safely. The powerful analytical capabilities of LLMs can be used to evaluate the effectiveness of these methods. The development of LLM-based intelligent tutoring tools and digital learning modules can further optimize teaching strategies and enhance the student learning experience, ultimately improving overall teaching quality.

Optimizing teaching content: to ensure that medical education remains aligned with the most up-to-date advancements in medical research and technology, researchers should regularly monitor and evaluate recent findings and clinical practices. These insights must be integrated into the curriculum via LLMs to ensure that the curriculum remains current and promotes continuous improvement.

While we have to identify certain fundamental principles and methods for an ethical framework for LLMs in medical education, several key areas require further refinement. First, this framework should be dynamic and adaptable to facilitate continuous updates as technology and society evolve. Second, a cross-cultural and global perspective is crucial with regard to respecting and accommodating ethical norms associated with different backgrounds. Additionally, involving a wide range of stakeholders—data providers, developers, policymakers, educators, students, and patients—is vital if their needs and concerns are to be reflected. Finally, strengthening ethical education and training is essential for stakeholders’ ability to understand and apply these principles. In the future, adhering to a unified ethical framework can establish a safe, transparent, fair, and high-quality environment to support the development of medical education.

#### Conclusions

This paper explores the complex challenges that LLMs may entail with regard to the future of medical education, and it covers the entire spectrum ranging from the development of such technologies to their practical application. It examines the limitations of current AI-related legal and ethical frameworks with regard to guiding LLM use in the context of medical education and advocates for the development of a unified ethical framework specifically for this purpose. This paper highlights 8 fundamental principles and detailed implementation measures necessary to create such a framework: privacy and data protection, data quality control and supervision, fairness and equal treatment, transparency and interpretability, academic integrity and moral norms, accountability and traceability, IP protection, and educational research and innovation. These principles provide a solid foundation for the development of a comprehensive and actionable ethical framework. Adhering to this unified ethical framework can offer clear guidance concerning the application of LLMs in medical education. Future research should refine and implement this ethical framework in the context of a rapidly evolving technological landscape, including by facilitating dynamic updates in light of new technologies, ensuring global cross-cultural applicability, engaging diverse stakeholders, and strengthening ethical education and training. These efforts aim to balance technological advancement with ethical values, thus, promoting progress in medical education without compromising the principles of fairness, equity, or patient safety with the goal of ensuring higher quality and greater inclusivity.

## Appendix

Multimedia Appendix 1 Artificial intelligence–related legal and ethical principles.

## Abbreviations

AI: artificial intelligence
IP: intellectual property
LLM: large language model
