# Supplementary material for: Ethical Considerations and Fundamental Principles of Large Language Models in Medical Education: Viewpoint
Source: J Med Internet Res. 2024 Aug 1;26:e60083. doi: 10.2196/60083 (PMC11327620; doi:10.2196/60083)
Supplement: Multimedia Appendix 1 [file jmir_v26i1e60083_app1.docx]

Multimedia Appendix 1. Artificial intelligence-related legal and ethical principles.

| **Date** | **Countries or organizations** | **Laws or ethical frameworks** | **Principles** |
| --- | --- | --- | --- |
| 2018-05 | European Union | General Data Protection Regulation | **Data security and privacy protection** |
|  |  |  | 1. Lawfulness, fairness and transparency |
|  |  |  | 2. Purpose limitation |
|  |  |  | 3. Data minimization |
|  |  |  | 4. Accuracy |
|  |  |  | 5. Storage limitation |
|  |  |  | 6. Integrity |
|  |  |  | 7. Confidentiality |
|  |  |  | 8. Accountability |
| 2021-08 | The People's Republic of China | Personal Information Protection Law | 1. Legitimacy |
|  |  |  | 2. Purpose |
|  |  |  | 3. Specification |
|  |  |  | 4. Minimal necessity |
|  |  |  | 5. Openness and transparency |
|  |  |  | 6. Accuracy |
|  |  |  | 7. Accountability |
|  |  |  | 8. Data security |
| 2019-01 | Institute of Electrical and Electronics Engineers | The IEEE Global Initiative on Ethics of Autonomous and Intelligent Systems (A/IS) | 1. Human Rights–A/IS shall be created and operated to respect, promote, and protect internationally recognized human rights. |
|  |  |  | 2. Well-being–A/IS creators shall adopt increased human well-being as a primary success criterion for development. |
|  |  |  | 3. Data Agency–A/IS creators shall empower individuals with the ability to access and securely share their data, with the aim of maintaining people’s capacity to have control over their identity. |
|  |  |  | 4. Effectiveness–A/IS creators and operators shall provide evidence of the effectiveness and fitness for the purpose of A/IS. |
|  |  |  | 5. Transparency–The basis of a particular A/IS decision should always be discoverable. |
|  |  |  | 6. Accountability–A/IS shall be created and operated to provide an unambiguous rationale for all decisions made. |
|  |  |  | 7. Awareness of Misuse–A/IS creators shall guard against all potential misuses and risks of A/IS in operation. |
|  |  |  | 8. Competence–A/IS creators shall specify and operators shall adhere to the knowledge and skill required for safe and effective operation. |
| 2021-11 | United Nations Educational Scientific and Cultural Organization | Recommendation on the Ethics of Artificial Intelligence | 1. Proportionality and do no harm |
|  |  |  | 2. Safety and security |
|  |  |  | 3. Fairness and nondiscrimination |
|  |  |  | 4. Sustainability |
|  |  |  | 5. Right to privacy and data protection |
|  |  |  | 6. Human oversight and determination |
|  |  |  | 7. Transparency and explainability |
|  |  |  | 8. Responsibility and accountability |
|  |  |  | 9. Awareness and literacy |
|  |  |  | 10. Multistakeholder and adaptive governance and collaboration |
| 2020-02 | European Union | White Paper on Artificial Intelligence—A European Approach to Excellence and Trust | **Four ethical principles** |
|  |  |  | 1. Respect for autonomy |
|  |  |  | 2. Prevention of harm |
|  |  |  | 3. Fairness |
|  |  |  | 4. Explainability |
|  |  |  | **Seven key elements for achieving trustworthy AI** |
|  |  |  | 1. Human agency and oversight |
|  |  |  | 2. Technical robustness and safety |
|  |  |  | 3. Privacy and data governance |
|  |  |  | 4. Transparency |
|  |  |  | 5. Diversity, nondiscrimination and fairness |
|  |  |  | 6. Societal and environmental well-being |
|  |  |  | 7. Accountability |
| 2024-01 | World Health Organization | Ethics and Governance of Artificial Intelligence for Health- Guidance on Large Multimodal Models | 1. Protect autonomy |
|  |  |  | 2. Promote human well-being, human safety and the public interest |
|  |  |  | 3. Ensure transparency, explainability and intelligibility |
|  |  |  | 4. Foster responsibility and accountability |
|  |  |  | 5. Ensure inclusiveness and equity |
|  |  |  | 6. Promote AI that is responsive and sustainable |
| 2024-03 | United Nations (Led by the United States） | Seizing the Opportunities of Safe, Secure and Trustworthy Artificial Intelligence Systems for Sustainable Development | 1. Cooperate with and provide capacity-building as well as technical and financial assistance to developing countries |
|  |  |  | 2. Bridge the AI divide and other digital divides that exist both between and within countries. |
|  |  |  | 3. Promote equitable access to the benefits of AI systems. |
|  |  |  | 4. Respect, protect, and promote human rights and fundamental freedoms throughout the life cycle of AI systems. |
|  |  |  | 5. Protect individuals from all forms of discrimination, bias, misuse, or other harm from AI systems. |
|  |  |  | 6. Develop regulatory and governance approaches and frameworks related to AI systems. |
|  |  |  | 7. Test AI systems prior to deployment and use. |
|  |  |  | 8. Raise public awareness of the appropriate civil use of AI systems. |
|  |  |  | 9. Encourage the development of tools that identify AI-generated digital content and their origin. |
|  |  |  | 10. Safeguard privacy and the protection of personal data. |
|  |  |  | 11. Respect intellectual property rights. |
|  |  |  | 12. Mitigate the potential negative consequences for workforces. |
|  |  |  | 13. Encourage the private sector to adhere to applicable international and domestic laws. |
